# Therapeutic outcomes of mycophenolate mofetil and glucocorticoid in thyroid-associated ophthalmopathy patients

**DOI:** 10.3389/fendo.2023.1140196

**Published:** 2023-03-21

**Authors:** Lan-Fang Li, Jun-Li Xue, Lei Guan, Fan-Fan Su, Hong Wang, Ding-Fu Zhang

**Affiliations:** ^1^ Department of Endocrinology, Jingzhou Cental Hospital, Jingzhou, China; ^2^ Department of Pediatrics, Jingzhou First People’s Hospital, Jingzhou, China; ^3^ Department of Ophthalmology, Jingzhou Cental Hospital, Jingzhou, China; ^4^ Department of Radiology, Jingzhou Cental Hospital, Jingzhou, China; ^5^ Department of Oncology, Jingzhou Cental Hospital, Jingzhou, China

**Keywords:** thyroid-associated ophthalmopathy, mycophenolate mofetil, glucocorticoid, therapeutic outcomes, adverse reactions

## Abstract

**Objective:**

To analyze the efficacy of mycophenolate mofetil (MMF) and glucocorticoid administration in patients with thyroid-associated ophthalmopathy (TAO).

**Methods:**

Sixty patients with moderate to severe TAO treated in Jingzhou Central Hospital from January 2022 to June 2022 were selected and enrtolled in this study. The subjects were divided into experimental group (n=30) and control group (n=30) based on the random number table method. Glucocorticoid pulse therapy was provided in the control group, while MMF was given in the experimental group on the basis of Control group. Clinical activity score (CAS), quality of life (QOL), visual acuity, eyelid fissure width, intraocular pressure, and degree of exophthalmos were observed at the time of admission and at the 12^th^ week and 24^th^ post-treatment weeks. We compared the immune function (TRAb, IL-6, and CD4+/CD8+) of the two groups pre-treatment and 24 weeks post-treatment, and evaluated the clinical therapeutic effect.

**Results:**

The clinical effective rates at 12 and 24 weeks in the experimental group were higher (73.3% and 83.3%) than those in the control group (46.7% and 60.0%) (*P <*0.05). After 12 weeks of treatment, patients’ CAS scores, and bilateral lid fissure width decreased and right eye visual acuity increased in the control group compared with those before treatment (*P <* 0.05); further, after 24 weeks of treatment, patients’ QOL scores and bilateral visual acuity increased and CAS scores, bilateral lid fissure width and proptosis decreased compared with those before treatment, and patients’ QOL scores, CAS scores and bilateral proptosis improved more than those at 12 weeks of treatment (*P <*0.05). Additionally, greater improvements were observed in the patients’ QOL and CAS scores, and proptosis after 24-week treatment than after 12-week treatment (*P*<0.05). In the experimental group, the QOL score and binocular visual acuity increased, whereas the CAS score, intraocular pressure, lid width, and proptosis decreased after 12 weeks of treatment as compared to the values of these parameters in the pre-treatment period (*P <* 0.05); after 24 weeks of treatment, greater improvements were established in the ocular-related indexes improved compared to the pre-treatment period and after 12 weeks of treatment (*P <* 0.05). After 12 weeks of treatment, the patients in the experimental group had more considerable improvements in the right visual acuity, right intraocular pressure, and left lid fissure width than the control group (*P <* 0.05); at 24 weeks of treatment, patients in the experimental group had greater improvements in the QOL score, bilateral visual acuity, intraocular pressure, bilateral lid fissure width, and bilateral proptosis than the control group (*P <* 0.05). No significant differences were found in the values of TRAb, IL-6, and CD4+/CD8+ between the two groups before treatment (*P*>0.05); the values of TRAb, IL-6, and CD4+/CD8+ in the experimental group was significantly lower than those before treatment and in the control group after 24weeks of treatment. (*P*>0.05). No statistically significant difference was observed in the incidence of liver damage and menstrual disorders between the two groups during the 24 weeks of treatment (*P*>0.05).

**Conclusion:**

The combination of oral MMF and glucocorticoid shock therapy is an effective drug for the treatment of patients with moderately active TAO.

## 1 Introduction

Thyroid-associated ophthalmopathy (TAO) is an organ-specific autoimmune, which is the most common orbital disorder in adults (1). The main clinical manifestations are soft-tissueedema, eyelid recession, proptosis, conjunctival congestion, and ocular motility disorders. The disease affects patients’ appearance, impairs visual functions, reduces their quality of life, causing significant psychological and financial burdens. In recent years, the incidence of TAO has been on the rise, but the cause and specific pathogenesis are still unclear, and thus no ideal treatment is available. Glucocorticosteroids are most commonly used for non-surgical treatment, but patients respond differently; no response is observed in some patients, whereas others respond only partially. MMF is a novel immunosuppressive agent that inhibits the differentiation of T lymphocytes, the synthesis of cell surface immune markers, and the activation of B lymphocytes. It is used mainly clinically to prevent rejection in renal and bone marrow transplant patients. In recent years, MMF has also been increasingly implemented in the treatment of autoimmune diseases. Studies at home and abroad have shown that MMF combined with methylprednisolone has good therapeutic effects in the treatment of TAO (2, 3). Therefore, in this study, to provide novel insights into clinical TAO treatment we aimed to investigate the therapeutic effects and safety of MMF administration combined with glucocorticoids in TAO patients.

## 2 Study subjects and methods

### 2.1 Study subjects

A total number of 60 patients with moderately severe active TAO, who were admitted to the Endocrine Department of Jingzhou Central Hospital from January 2022 to June 2022, were selected. The following inclusion criteria were applied: (1) age of 18–70 years, male and female patients; (2) TAO diagnostic criteria (4) were met for both eyes; (3) the patients’ eye disease degree was moderately severe according to the grading criteria of eye disease severity proposed by the European Group of Experts on Graves’ Ophthalmology (EUGOGO) consensus in 2016 (5); (4) mean CAS score ≥ 3 in both eyes according to the active stage clinical activity score (CAS) (5); (5) an informed consent form was signed. The exclusion criteria implemented in this study were as follows: (1) contraindications to glucocorticoid therapy, including peptic ulcer, active hepatitis, tuberculosis, presence of uncontrolled hypertension and diabetes, severe osteoporosis, severe infection, hepatic and renal insufficiency, glaucoma, psychiatric disorders, and glucocorticoid allergy; (2) pregnant and lactating women; (3) pre-existing ocular pathology, such as retrobulbar space-occupying disease, hypertensive eye disease, or diabetic retinopathy; (4) a history of previous ocular surgery such as orbital decompression or eyelid surgery: (4) other autoimmune diseases, malignant tumors; (5) immunomodulatory or cytotoxic drug therapy applied within three months.

A total number of 60 patients were enrolled and divided into a control group (n = 30) and an experimental group (n = 30) using the random number table. The study was approved by the ethics committee of the hospital, and informed consent was signed before the hormone shock and MMF oral treatment.

### 2.2 Study methods

Of the 60 patients, 32 had hyperthyroidism, 16 hypothyroidism, 3 subclinical hyperthyroidism, 3 subclinical hypothyroidism, and 6 normal thyroid function, 5 of whom had proptosis after 131I treatment. We followed up the patients regularly and gave anti-thyroid drugs to those with accompanying hyperthyroidism and levothyroxine tablets to those with accompanying hypothyroidism, and adjusted the dose based on the FT3, FT4, and TSH levels to maintain stable thyroid functions. Both groups were given conventional symptomatic treatment and instructions (4): to quit smoking; to follow a low-salt and low-fat diet; to avoid prolonged eye use; to wear dark glasses outside; to use artificial tears, gel, or eye ointment to protect the cornea; to ensure they obtain adequate sleep. The control group was given methylprednisolone shock therapy, methylprednisolone 500mg + saline 250 mL intravenously once a week, six times, followed by methylprednisolone 250mg + saline 250 mL intravenously once a week, six times. In the experimental group, 0.25 g of oral MMF (Saikopin, Zhongmei Huadong Pharmaceutical Co., Ltd) was added to the treatment applied in the control group, 0.36g twice daily for 24 weeks. During the period of methylprednisolone treatment, we also administered calcium and potassium supplementation as symptomatic treatment for gastric acid suppression and protection.

### 2.3 Observational indicators

The patients’ CAS score, QOL (quality of life) score (5), lid fissure width, proptosis, visual acuity, intraocular pressure, soft-tissue inflammation, and diplopia status were observed at the time of enrolment, week 12, and week 24. The same ophthalmologist measured proptosis with a Hertel exophthalmometer, visual acuity with an international standard visual acuity chart, intraocular pressure with a non-contact tonometer, and the lid slit width of the primary eye position.

(1) Effective treatment: At least two or more of the following indicators were to be met, and none of the indicators should not have deteriorated: ①reduction in the CAS score by ≥2 points; ②reduction in the lid width by ≥2 mm; ③reduction in proptosis by ≥2 mm; ④ improvement by ≥1 grade in the soft-tissue involvement, such as eyelid or conjunctival redness and eyelid or conjunctival edema; ⑤reduction in the diplopia grade by ≥1 grade;
(2) Worsening: At least two or more of the following criteria were to be met: ①≥2 points increase in the CAS score; ②≥2 mm increase in the lid width; ③≥2 mm increase in proptosis; ④≥1 level of worsening of any of the soft-tissue involvements, such as eyelid or conjunctival redness and eyelid or conjunctival edema; ⑤≥1 level of increase in diplopia;
(3) Unchanged: no change in the above indicators or a change of less than the aforementioned defined values (6). Before treatment and at 24 weeks of treatment, the TRAb and IL-6 levels were measured by electrochemiluminescence method, and the CD4+/CD8+ ratio was measured by flow cytometry in both groups and compared.

Throughout the trial, we had a dedicated member in charge of regular telephone and outpatient follow-up consultations that counted, recorded, and compared the incidence of adverse reactions during the treatment in both groups. For example, the incidence of common adverse reactions to glucocorticoids and mycophenolate esters were observed, such as nausea and vomiting, liver function damage, kidney function damage, reduction in the white blood cell count, blood sugar levels increase, menstrual disorders, infection, and other related adverse reactions. No patients dropped out of the group during the entire study process, with a 100% follow-up rate.

### 2.4 Statistical analysis

SPSS 17.0 was used for statistical analysis. Measurement data were expressed as χ ± s, and the normal distribution t-test was used for intra-group and inter-group comparisons. Count data were expressed as cases (%), χ2 test was employed for inter-group comparisons. *P <* 0.05 was considered to indicate a statistically significant difference.

## 3 Results

### 3.1 Comparison of general information between the two groups

No statistically significant differences were found between the two groups in terms of gender, age, duration of TAO, CAS score, TAO-QOL score, TRAb, IL-6, and CD4+/CD8+(*P*>0.05; **Table 1**).

**Table 1:** Comparison between the general information data of the two groups.

Group	N	Gender (M/F)	Age (years)	Duration of TAO (months)	QOL score	CAS score	TRAb	IL-6	CD4+/CD8+
Control group	30	12/30	41.47 ± 9.83	11.67 ± 5.13	66.52 ± 23.00	4.40 ± 0.81	16.53 ± 9.41	11.55 ± 7.04	3.09 ± 1.48
Experimental group	30	8/30	44.13 ± 11.69	11.00 ± 4.69	61.88 ± 20.64	4.23 ± 0.90	17.38 ± 9.52	14.26 ± 7.34	2.50 ± 1.17
P		0.281	0.34	0.62	0.42	0.45	0.73	0.15	0.09

### 3.2 Comparison of the treatment efficiency in the two groups

The clinical efficiency of the experimental group was higher than that of the control group at 12 and 24 weeks, with a statistically significant difference (*P*<0.05; **Table 2**).

**Table 2:** Comparison of effective rates between the two groups.

Time	12 weeks				24 weeks			
Subgroup	Control group	Experimental group	χ^2^	*P*	Control group	Experimental group	χ^2^	*P*
N	30	30			30	30		
Valid	14	22			18	25		
Unchanged	14	7			11	5		
Deteriorated	2	1			1	0		
Effective	46.7%	73.3%	4.44	0.04	60.0%	83.3%	4.02	0.04

### 3.3 Comparison of the indicators between the two groups before treatment, at 12 weeks, and at 24 weeks of treatment

After 12 weeks of treatment in the control group, the patients’ CAS scores and binocular lid width decreased, and right eye visual acuity increased as compared to the values determined before the treatment (*P <* 0.05). After 24 weeks of treatment, patients’ QOL scores and binocular visual acuity were higher than those established before the treatment; the CAS scores, binocular lid width, and proptosis were lower than those measured before the treatment. The patients’ QOL scores, CAS scores, and binocular proptosis improved better than at 12 weeks of treatment (*P <* 0.05) than at 24 weeks. In the experimental group, after 12 weeks of treatment, patients’ QOL scores and binocular visual acuity increased, and CAS scores, binocular pressure, lid width, and proptosis decreased compared to those before the treatment (*P <* 0.05). After 24 weeks of treatment, patients’ eye-related indexes improved compared with those before the treatment and at 12 weeks of treatment (*P <* 0.05). At 12 weeks of treatment, the right visual acuity, right binocular pressure, and left lid fissure width improved more in the experimental group than in the control group (*P <* 0.05). After24-week treatment, greater improvements were observed in the QOL score, bilateral visual acuity, intraocular pressure, bilateral lid fissure width, and bilateral proptosis in the experimental group than in the control group (*P <* 0.05; **Table 3**).

**Table 3:** Comparison of indicators between the two groups before the treatment, at 12 weeks, and at 24 weeks of treatment.

Subgroup	Control group			Experimental group			
	Pre-treatment	12 weeks	24 weeks		Pre-treatment	12 weeks	24 weeks
QOLscore	66.52 ± 23.00	72.06 ± 16.23	80.12 ± 11.75^ab^		61.88 ± 20.64	77.34 ± 9.58 ^a^	87.90 ± 6.23^abc^
CASscore	4.4 ± 0.81	3.00 ± 0.91^a^	2.13 ± 0.94^ab^		4.23 ± 0.90	2.90 ± 1.00 ^a^	1.83 ± 0.83 ^ab^
Visual acuity (right mm)	0.63 ± 0.18	0.73 ± 0.16^a^	0.74 ± 0.15^a^		0.63 ± 0.22	0.81 ± 0.16 ^ac^	0.90 ± 0.10 ^abc^
Visual acuity (left mm)	0.64 ± 0.18	0.73 ± 0.16	0.77 ± 0.14^a^		0.67 ± 0.24	0.81 ± 0.19 ^a^	0.91 ± 0.17 ^abc^
Intraocular pressure (right mm)	21.43 ± 3.96	20.33 ± 1.73	20.10 ± 1.49		20.22 ± 3.29	18.37 ± 1.90 ^ac^	17.17 ± 1.78^abc^
Intraocular pressure (left mm)	20.92 ± 4.09	19.67 ± 2.32	19.40 ± 2.08		20.27 ± 3.20	18.80 ± 1.97 ^a^	17.70 ± 1.09 ^abc^
Lid fissure width (right mm)	11.07 ± 2.16	9.90 ± 1.54^a^	9.98 ± 1.58^a^		10.77 ± 2.31	9.57 ± 1.34 ^a^	8.47 ± 1.07 ^abc^
Lid width (right mm)	11.60 ± 1.9	10.27 ± 1.41^a^	10.67 ± 1.75^a^		11.03 ± 2.11	9.57 ± 1.43 ^ac^	8.47 ± 1.14 ^abc^
Proptosis (right mm)	20.73 ± 2.05	19.87 ± 1.61	19.00 ± 0.98^ab^		20.77 ± 2.82	19.13 ± 2.03 ^a^	17.63 ± 1.16^abc^
Proptosis (right mm)	20.35 ± 2.34	19.47 ± 1.28	18.77 ± 1.17^ab^		20.70 ± 2.18	19.00 ± 1.64 ^a^	18.03 ± 1.16 ^abc^

Compared to pre-treatment, ^a^P < 0.05 , compared to 12 weeks of treatment, ^b^P < 0.05 ; compared between the two groups at 12 weeks and 24 weeks of treatment, ^c^P < 0.05.

### 3.4 Comparison of the immune function between the two groups pre- and post-treatment

There was no statistically significant difference in the levels of TRAb, IL-6, and the CD4+/CD8+ ratio between the two groups before the treatment (*P*>0.05). At 24 weeks of treatment, the TRAb and IL-6 levels and the CD4+/CD8+ ratio in both groups were lower than those before the treatment, with a more significant decrease in the experimental group (*P*<0.05; **Table 4**).

**Table 4:** Comparison of the immune function between the two groups of patients pre- and post-treatment.

Subgroup	Time	TRAb	IL-6	CD4+/CD8+
Control group	Before treatment	16.53 ± 9.41	11.55 ± 7.04	3.09 ± 1.48
	24 weeks	7.17 ± 3.51^a^	8.32 ± 3.95^a^	2.25 ± 0.92^a^
Experimental group	Before treatment	17.38 ± 9.52	14.26 ± 7.34	2.50 ± 1.67
	24 weeks	4.46 ± 2.99^ab^	6.22 ± 2.94^ab^	1.60 ± 0.50^ab^

Compared to pre-treatment, ^a^P < 0.05; 24 weeks post-treatment, ^b^P < 0.05 in the experimental group compared to the control group.

### 3.5 Occurrence of adverse reactions

No more significant adverse reactions occurred in either group after the administration of the drug. In the control group, there were two cases (6.7%) of mild abnormal liver function and two cases (6.7%) of menstrual disorders in the control group. In the experimental group, we observed three cases (10%) of mild abnormal liver function and one case (3.33%) of menstrual disorders. However, the differences were not statistically significant (χ2=1.22, *P*> 0.05).

## 4 Discussion

The pathogenesis of TAO is not yet fully elucidated and is now generally accepted to be the result of the action of a combination of cellular and humoral immunity factors under different genetic and environmental conditions. Glucocorticoids inhibit the release of cytokines, interfere with the function of T and B lymphocytes, and reduce the proliferation of orbital fibroblasts; hence, they are the main modality for the treatment of moderate-to-severe TAO (7). Long-term high doses of glucocorticoids can cause systemic adverse effects, such as increased blood glucose and blood pressure, decreased bone mass due to osteoporosis, gastric ulcers, and infections. Some patients do not respond to glucocorticoid treatment (8).

MMF is a 2-ethyl ester derivative of mescaline, which blocks the de novo synthesis pathway of guanine nucleotides by inhibiting the hypoxanthine mononucleotide dehydrogenase inosine 5′-monophosphate dehydrogenase (IMPDH). Additionally, MMF decreases the level of guanosine triphosphate and selectively inhibits the differentiation proliferation and the activation of T and B lymphocytes, and induces their apoptosis, thereby inhibiting antibody production and other effector cell responses. Furthermore, MMF also suppresses the expression of vascular cell adhesion molecule 1 (VCAM-1) on the surface of lymphocytes and the aggregation of lymphocytes and monocytes at the site of inflammation, thus strongly inhibiting the proliferation of T and B lymphocytes and exerting immunosuppressive effects (9). In addition, MMF has important anti-inflammatory effects. MMF suppresses the action of specific pro-inflammatory enzymes and reduces the mRNA and protein expression levels of tumor necrosis factor-α, interleukin β, and vascular endothelial growth factor, thereby reducing the inward flow of leukocytes, decreasing the concentration of inflammatory exudates, and exerting anti-inflammatory effects (10). Previous research has shown that MMF is highly specific for the treatment of TAO (11). MMF was found to be significantly more effective than prednisone in the treatment of TAO, with fewer adverse effects (12). In this study, after 12 and 24 weeks of treatment, the clinical efficiency of oral MMF combined with glucocorticoid shock treatment was higher (73.3%, 83.3%) than that of the glucocorticoid shock treatment alone (46.7%, 60.0%), suggesting that the clinical efficiency of the combination therapy of MMF and glucocorticoid TAO treatment was higher than that of glucocorticoid treatment alone.

In this study, the patients treated with glucocorticoid shock therapy alone resulted in improvements in the CAS scores, left eye visual acuity, and bilateral lid fissure width at 12 weeks, but not in the QOL scores, intraocular pressure, and proptosis. Glucocorticoids can reduce periorbital tissue inflammation and improve patients’ quality of life, but have no significant effect on intraocular pressure. The oral combination of MMF with glucocorticoid shock treatment improved the CAS score, QOL score, visual acuity, intraocular pressure, lid fissure height, and proptosis of the patients at 12 weeks of treatment. These parameters further improved after 24 weeks of treatment. At 24 weeks of treatment, the QOL score, binocular visual acuity, binocular pressure, bilateral lid fissure width, and bilateral proptosis of the patients in the experimental group improved more than those in the control group (*P <*0.05), indicating that the combination of MMF and glucocorticoids in the treatment of TAO can achieve better clinical results than intravenous shock with glucocorticoids alone. The pathological mechanism of TAO is exerted by the stimulation of fibroblasts by T and B lymphocytes and multiple cytokine-mediated immune responses, which promote the accumulation of hyaluronic acid, mucopolysaccharide and glycosaminoglycan (GAG) in the orbit, triggering proptosis and extraocular muscle damage (13). MMF selectively inhibits the differentiation proliferation and activation of T and B lymphocytes, induces their apoptosis, and suppresses the inflammatory response, which may be the reason for further improvement of proptosis after the addition of MMF.

Activated B lymphocytes in TAO patients produce antibodies against the thyroid-stimulating hormone receptor TSHR (TSHR-stimulating antibodies), causing a periorbital inflammatory response.IL-6 promotes the proliferation and differentiation of B cells and T cells, releasing a variety of cytokines and leading to increased production of glycosaminoglycans and fat in the orbit. T lymphocytes express CD3 molecules and can be divided into CD4+ T lymphocytes and CD8+ T lymphocytes depending on whether they express CD4 and CD8 molecules on their surface (14).Activated CD4+ T lymphocytes produce a variety of adhesion molecules that, together with chemokines and adhesion molecules stimulated by orbital fibroblasts, mediate the entry of more lymphocytes into the orbital tissue and mediate the inflammatory response in TAO (15).

Our present results show that the TRAb andIL-6, levels and the CD4+/CD8+ ratio were lower than those before the treatment, regardless of whether they were treated with glucocorticoid shock therapy alone or with glucocorticoid shock therapy in combination with oral MMF administration. Their decrease was more pronounced in the combination treatment than in the glucocorticoid treatment alone, which suggests that the combination of glucocorticoids and MMF improves the condition of TAO patients by suppressing immune pathways. The combination of MMF did not increase the number of significant adverse effects.

Previously, the first-line treatment regimen for TAO was hormone shock therapy, but beneficial effects were achieved only in part of patients. Recently, the 2021 EUGOGO clinical practice guidelines included mycophenolate mofetil in the first-line treatment regimen for TAO. However, not many relevant reports have been published in China. Mycophenolate mofetil has not been widely applied for the treatment of Chinese TAO patients, and hence there is a lack of reports on its efficacy and adverse effects. In our study, the combination of oral MMF and glucocorticoid shock therapy was found to improve the therapeutic efficiency with low numbers of adverse effects. Therefore, the combination of oral MMF and glucocorticoid shock therapy is an effective drug for the treatment of patients with moderately active TAO. However, future prospective multicenter randomized controlled studies with more cases need to be performed to further evaluate the efficacy and safety of MMF in patients with active moderate-to-severe TAO.

## Data availability statement

The original contributions presented in the study are included in the article/supplementary material. Further inquiries can be directed to the corresponding author.

## Ethics statement

The studies involving human participants were reviewed and approved by Yijun Ren. The patients/participants provided their written informed consent to participate in this study.

## Author contributions

The study was designed by J-LX and LF-L, and data extraction and analysis were collected by L-FL and LG. The initial manuscript was written by L-FL and finally revised by J-LX and LG. L-FL, F-FS, HW and D-FZ were involved in the return visit and data collection of patients. All authors contributed to the article and approved the submitted version.
